# Citizen participation in climate politics. Drivers and barriers of Climate Assemblies in Europe

**DOI:** 10.12688/openreseurope.21452.1

**Published:** 2026-01-02

**Authors:** Erich Griessler, Maria Alonso Raposo, Lucia Cristea, Floridea Di Ciommo, Elisabeth Frankus, Liliana Denisa Andrei, Shauna Stack

**Affiliations:** 1 Socials Sustainable Transformation, Institut fur Hohere Studien-Institute for Advanced Studies, Vienna, Vienna, 1080, Austria; 2 Cambiamo, Madrid, 28012, Spain; 3 European Integrated Projects, Bucarest, 080116, Romania

**Keywords:** Climate change, climate adaptation, climate policy, citizen participation, climate assemblies, Europe, deliberative democracy, political culture

## Abstract

**Background:**

Different forms of participation have been employed to engage citizens in the planning of climate change mitigation and adaptation strategies. Arguments in favor of citizen participation highlight the limitations of traditional democratic practices to address climate change. Climate Assemblies (CAs), a form of deliberative democracy, have become an increasingly popular way for citizens and politicians to collaborate on climate decision-making.

**Research Questions:**

Using a mixed methods approach, this paper poses three questions. (1) To what extent do European cities and regions engage in CAs, and how are they embedded in policymaking? (2) What drives and impedes CAs? (3) To what extent are policymakers in European cities and regions ready and able to incorporate CAs and their results into policies?

**Results:**

Findings reveal an increase in CAs in Europe on different levels, primarily commissioned by public authorities. However, the connection between CAs and policymaking differs across countries. Research revealed the significance of political culture, the specific roles of citizens, policymakers and administration therein, and the importance of political backing of CAs. Important drivers of CAs include measures that safeguard relevance to citizens, equality, inclusive access, and impact. Barriers include knowledge about climate change and deliberative democracy, lacking inclusiveness of CAs and asymmetry in political power. Survey data shows that climate policies have become established practices in many European cities and regions and that various engagement approaches are used to develop them. However, only 9.4% of respondents stated that city officials developed climate change policies with stakeholder input, including citizens. Citizen participation is infrequent, and involvement in policy development and implementation is unequally distributed, favoring some groups over others. While some results of stakeholder and citizen engagement activities were adopted, recommendations were not always translated into policies.

**Conclusions:**

Currently, CAs are rather an exception than the norm across Europe.

## Plain Language Summary

There are various ways to engage citizens in the development of climate policies. Supporters of public involvement in these issues argue that traditional democratic processes are inadequate for addressing climate change and mitigation. One way people and politicians collaborate in this area is through a Climate Assembly (CA). At such an event, a group of citizens chosen at random from different backgrounds come together to learn about and discuss ways to tackle climate change. Then, they provide recommendations to policymakers. This paper asks three questions: (1) How often do European cities and regions use CAs, and how are they used to develop policies? (2) What enables or prevents CAs from happening? (3) How ready are policymakers to use CAs and their results? The results show that the number of CAs in Europe is increasing, often on behalf of public authorities. However, the relationship between CAs and policymaking varies. The research demonstrated the importance of political culture and support. Key factors of CAs include ensuring that topics are relevant to citizens, that everyone is treated equally and has equal access to CAs, and that CAs have an actual impact on policies. Three main barriers exist: First, when citizens and policymakers lack sufficient knowledge about climate change and deliberative democracy, Second, when CAs are not inclusive enough. (3) When political power is not distributed equally between citizens and policymakers. The survey shows that many European cities and regions have climate policies and use different methods to develop them. However, only 9.4% of respondents from European cities and regions said city officials developed climate change policies with stakeholder input, including from citizens. While some citizen engagement was used, their recommendations were not always turned into policies. CAs are the exception rather than the norm in Europe.

## Introduction

Various forms of public participation have been used to involve citizens in planning to mitigate or adapt to climate change, ranging from surveys and focus groups to future visioning workshops and green hackathons ( Galende-Sánchez & Sorman, 2021; OECD, 2020a; Stack *et al*., 2023). The argument often used to support citizen participation in this area points to limitations of traditional democratic practices to address climate change, necessitating the exploration of deliberative practices as both an experiment in democratic renewal and a response to the climate emergency ( Curato *et al*., 2022). Activists and scholars have argued that citizen engagement is imperative due to the political responses, or lack thereof, to the climate emergency. Reasons for this are reflected in the mismatch between the long-term nature of climate change and the shorter-term pressures of election cycles and lobbying campaigns, as well as the power imbalance with politically savvy actors and citizens whose voices might be weaker in comparison. Consequently, the current democratic system lacks incentives for the substantial challenges and investments essential for long-term climate change adaptation and planning ( Gupta *et al.*, 2007).

Since the 1960s advocates of deliberative democracy, which is a form of democracy in which various stakeholders and citizens deliberate about a topic as part of the decision-making process, have been experimenting with different formats such as planning cells ( Dienel, 1999), citizens’ juries, citizens’ panels, and consensus conferences ( Courant, 2021a; Courant, 2021b; Devaney *et al.*, 2020; Fung, 2003). Mansbridge (2017) describes the democratic advantages of deliberation as “recursive representation”, which involves fostering two-way interactions between politicians and citizens that go beyond established democratic practices such as voting. This higher-order form of engagement promotes mutual learning and understanding of diverse views, values and potential actions between political representatives and the citizens who elect them. “Deliberative mini publics” are a specific type of deliberative format that takes a randomly selected representative sample of the wider population to deliberate about and provide thoughtful input (usually in the form of considered policy recommendations) to a particular topic ( Setälä, 2017). Depending on size, structure, and time allocated for the process, these formats are named differently, e.g., citizen juries or citizen assemblies. Applied to climate policy, citizen assemblies are usually referred to as Climate Assemblies (CAs) and have been recently increasingly used as a pathway for citizens and politicians to work together on climate decision making ( OECD, 2020b; OECD, 2021; OECD, 2025). They stand out as sustainable and effective tools for promoting deliberative democracy in climate policy making ( Elstub & Escobar, 2017) and are characterized by the gathering of a randomly selected but diverse group of citizens to engage in a structured learning and deliberation process to produce recommendations about how to respond to climate emergencies and adaptation ( Cherry *et al*., 2021).

As an innovation in democracy, CAs promise to improve policy making by involving ordinary citizens into the development of policies for climate change mitigation and adaptation they are a democratic innovation in the established political system and in this way challenge roles, self-understanding, relationships of citizens, politicians, civil-servants, civil society organizations (CSOs) and media in policy making. This paper looks at CA as a policy innovation and poses three questions. (1) To what extent do European cities/regions engage in CAs, and how are they embedded in policy-making processes? (2) What drives and impedes CAs? (3) To what extent are policymakers in European cities and regions ready and able to incorporate CA and their results into policy making and policies?

The paper starts by describing the applied methods and then outlines the current state of play in European CAs. This is based on mapping the strategies of individual CAs through desk research, as well as determining, differentiating and contextualizing these factors. The next section presents data collected during stakeholder workshops on the barriers and drivers of integrating CAs into policy-making processes. The following section distils insights from survey results collected from local European politicians and policymakers regarding their experiences with citizen engagement activities. Finally, we conclude by integrating these three mixed-methods data sources to provide actionable insights for scaling up CAs, particularly for those in a position to initiate, support, fund and drive democratic participatory processes in their communities.

## Methods

This paper is based on triangulation of qualitative and quantitative research methods: Desk research mapped CA exercises across Europe to address the first research question; a series of participatory stakeholder workshops identified drivers and barriers of CAs (research question 2); a survey explored current practices, experiences and infrastructure of European cities and regions with citizen engagement in climate policies (research question 3).

### Mapping CAs in Europe

To identify CAs in Europe, systematic desk research was conducted in the spring and summer of 2023. This covered 308 local communities and nations that had signed the EU Mission on Adaptation to Climate Change ( European Commission, 2025). To qualify, a CA had to have a website, blogpost, or some form of online representation. Databases for participatory democracy, such as KNOCA (n.d.); Participedia (n.d.); the German Bürgerrat (n.d.) were consulted for validation. ^1^ However, since not all local CAs could be validated there, cross-verification from websites and independent news sources was necessary.

While identifying CAs was straightforward, delving into the details proved more challenging. ^2^ This was primarily because the prevailing practice of presenting CAs in reports emphasized outcomes in terms of recommendations rather than process nuances. Websites often lacked descriptions of the actual experiences within the CA, hindering a comprehensive understanding of their unique qualities. In addition, in several cases, information about the CA was only available in the aforementioned databases that collect instances of CAs. In these cases, information about the CA was less detailed. In most cases, however, CAs provided a web page and more detailed information. In many cases, recommendations, reports, invitation letters, information leaflets, and minutes were available. Final reports and recommendations from the CA included, in many cases, also information about the CA process, and, in a few instances, short evaluations. Independent expert evaluations were only available in a few cases.

In addition, a rapid review of literature was carried out in 2023, using Google Scholar, incorporating research papers and grey literature. Boolean search operators were used such as : “agenda setting” AND “climate assembly”, “engagement” AND “deliberation” AND “climate assembly”, “inclusion” AND “methods” AND “climate assembly”, “digital tools” AND “climate assemblies”, “follow up” AND “climate assembly”, “voting” AND “deliberation” AND “climate assembly”, “mini public” AND “climate assembly”. This generated about 150 papers, which underwent screening for relevance, with approximately 50 papers deemed informative for our question. Scholarly literature aided in the identification and, sometimes, enrichment of case studies identified in the mapping.

Websites and related documents of CAs were qualitatively analyzed by thematic analysis ( Froschauer & Lueger, 2003). If information was only available on government and project websites, as well as on websites that collect participatory actions, data sheets were created that included organizational details such as country, government level, date and duration of CA as well as information about input, process, output, outcome and impact. No such data files were created when more detailed material was available, such as reports, recommendations, government responses, pamphlets, fact sheets, letters, evaluations, and journal articles. This material was uploaded to the database, which ultimately contained 134 documents from 74 cases. Qualitative analysis started with familiarizing with the material and continued with open coding, resulting in 89 codes being generated inductively from the documents.

### Stakeholder workshops

**Figure 1.**
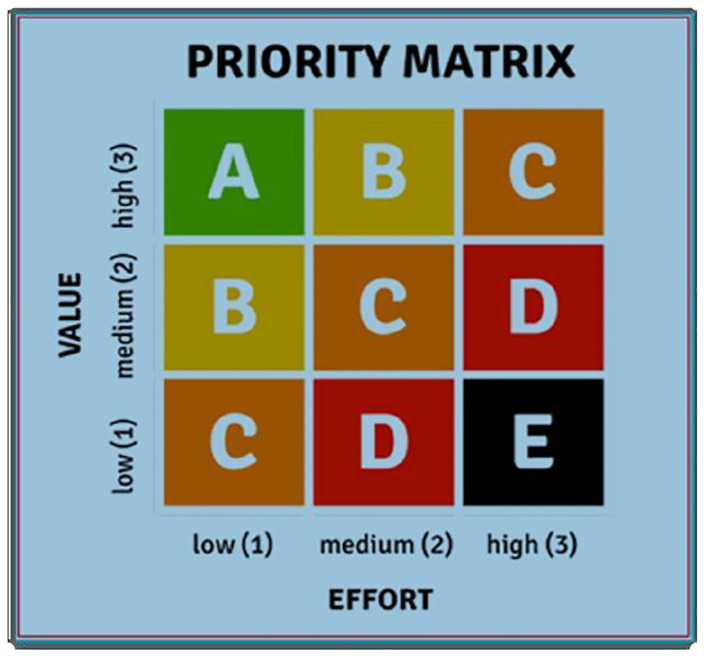
Prioritization table. Source: Di Ciommo *et al.* (2023).

Three online workshops were held between May and September 2023 to identify drivers and barriers of CA. The 21 male and 22 female attendants had experience with organizing CAs and/or deliberative decision-making processes, as well as with climate change and adaptation. They came from academia (15), citizen advocacy networks (14), civil society (9), as well as policymaking (5). Methods used were open discussion and working on real-time collaborative web platforms to collect views and experiences about bottlenecks, barriers and drivers for deliberation in CA. Consensus-building, and prioritization methods were used to identify key factors that influence the implementation of CAs ( Figure 1). For that, participants ranked topics as “low”, “medium” or “high” according to their (1) relevance and (2) the effort necessary to overcome the obstacle. This resulted in prioritization ranging from A to E.

### Survey

Between October and November 2023, an English language, online survey was sent to 450 contacts in 140 European cities and regions to collect data on practices and capacities of citizen engagement of European municipal and regional authorities in policy- and decision-making processes on climate change. In addition, the survey was disseminated in 2023 during the Urban Mobility Days in Sevilla and the Smart City Expo World Congress in Barcelona, which were thematically related to issues of climate change mitigation and adaptation. The survey explored (1) the status of climate change policy development in European cities/regions; (2) barriers European cities/regions face or drivers which facilitate the development/implementation of climate change policies; (3) practices and capacities to engage with stakeholders ^3^, including citizens; (4) whether public authorities organize CA and if not, why.

The final sample comprised of 65 received and 64 validated responses ( Andrei & Cristea, 2023). After eliminating the incomplete responses, frequencies were analyzed. The main answers to the survey came from medium to large urban areas, while smaller municipalities and rural areas were particularly under-represented. Despite these limitations, the CLIMAS survey provides essential insights into the landscape of stakeholder engagement in climate policymaking across Europe. The findings emphasize the demand for thorough analysis and particular methodological improvements.

## Results

### Mapping climate assemblies

**Figure 2.**
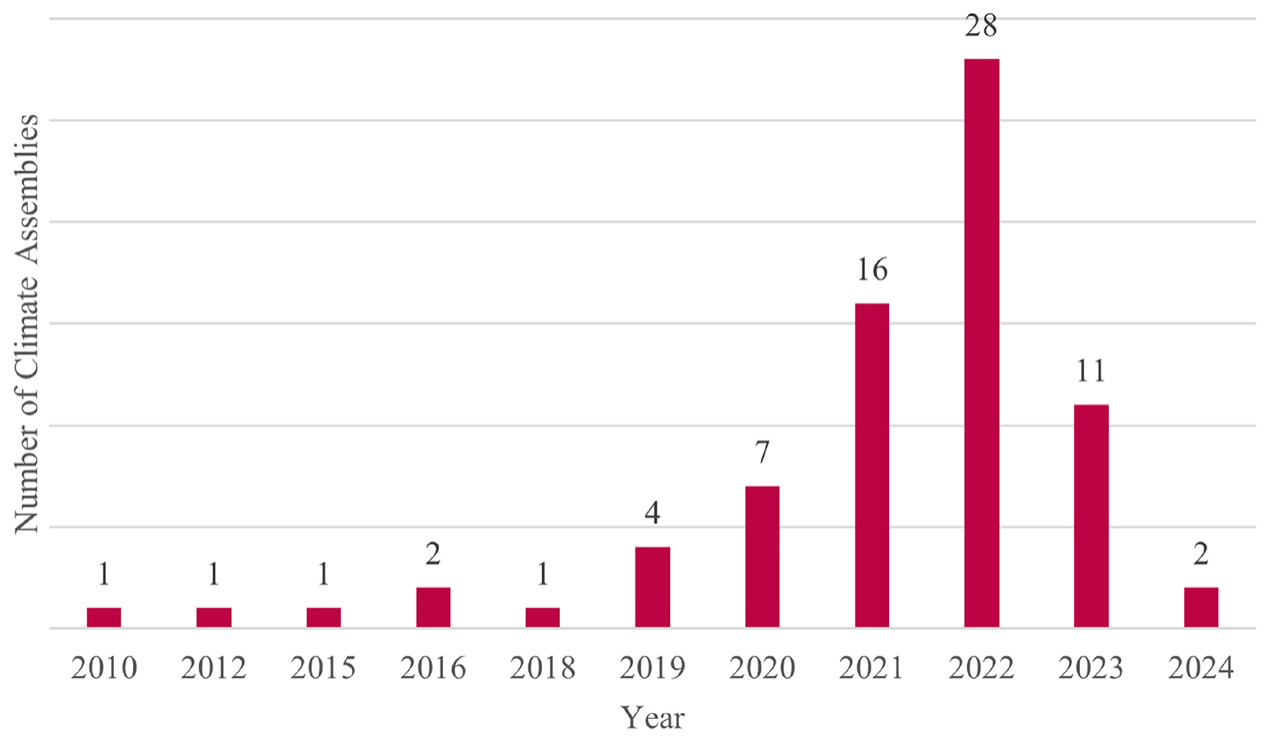
Selected Climate Assemblies by year. Source: Stack *et al.* (2023).

Mapping resulted in a sample of 74 CAs that were organized between 2010 and 2024. Numbers of CA increased in 2021 and 2022 before decreasing again in 2023 ( Figure 2).

**Figure 3.**
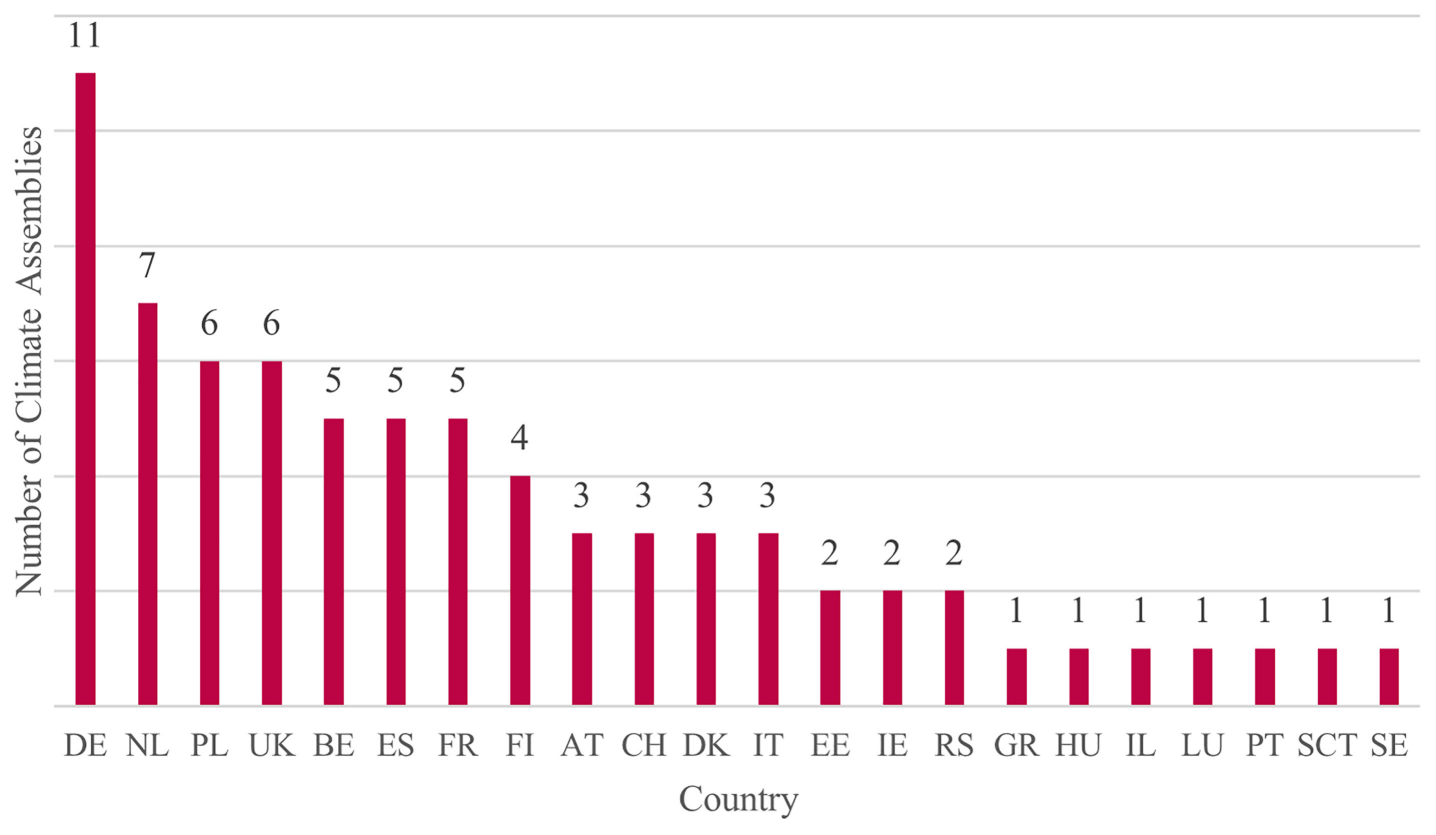
Selected Climate Assemblies by country. Source: Stack *et al.* (2023).

Figure 3 shows distribution of CAs in the sample across Europe. CAs included were more prevalent in some countries than in others. The largest numbers of CAs were situated in Germany, followed by the Netherlands, Poland and the UK. ^4^

CAs were organized on different government levels, eleven CAs were organized at a national level (15%), 16 at a regional level (22%), and 47 at a local level (63%). Numbers of participants varied between levels. National CAs generally were the largest with a number of participants between 100 and 200, whereas regional CAs generally had fewer participants, ranging from 20 to 60. Local CAs generally had fewer participants, ranging from 20 to 50, but there were also a few with up to 150 participants.

**Figure 4.**
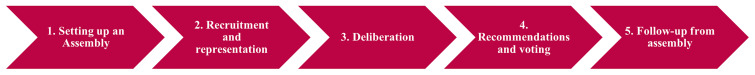
Phases of a Climate Assembly. Source: OECD, 2023; Smith, 2023 adaptation by Stack *et al.* (2023).

A CA has several phases (e.g., Di Ciommo, 2023; OECD, 2020a; Smith, 2023; Stack *et al*., 2023) including (1) setting up the CA, (2) recruitment and representation, (3) deliberation, (4) recommendations and voting, and (5) CA follow-up ( Figure 4).

Although these steps must be accustomed to specific cultural, social, political and environmental contexts, CAs face shared challenges such as considering the design, implementation, governance, and utilization of participatory methods ( Courant, 2021a; Courant, 2021b; Lewis *et al*., 2023; Pow, 2023) as well as their embedding in the political context to ensure legitimacy and impact. Several handbooks offer guidance on the stages of a CA ( Extinction Rebellion, 2019; Involve, n.d.; KNOCA, 2022a; OECD, 2020b; People Powered, 2020; The Democratic Society, n.d.). These guidebooks have established a well-defined practice for recruitment, deliberation, recommendations and voting. This paper focuses on the embeddedness of CAs in the political system which plays out in three phases: (1) at the start of the CA process, in terms of who initiated the event; (2) during the CA process, if policymakers participate in this phase; (3) at the end of the process and thereafter, in terms of to what extent authorities adopt recommendations.

At the start, commissioners initiate and sponsor a CA. In this respect, “political coupling” ( Hendriks, 2016) — that is, the commissioner's role and relationship with the political system and civil society is crucial ( KNOCA, 2022b). Mapping revealed three ways of commissioning a CA: (1) “top-down” by local, regional, or national authorities; (2) “bottom-up” by CSOs engaged in combating climate change and (3) “hybrid”, in a joint initiative of public authorities and CSOs. Political coupling is associated with a trade-off between legitimacy and impact. Research suggests that CAs originating from politics are more likely to generate political impact and engagement ( Niessen, 2019), whereas grassroots initiatives may be viewed by the political system as uninvited and unwanted interventions that interfere with representative democratic processes. “Top down” and “hybrid” may increase legitimacy and ownership within the political system, thus facilitating the adoption of their outcomes. Conversely, “bottom up” may increase CAs’ independence from politics, reducing the risk of it being perceived as co-opted by political authorities. On the other hand, bottom-up initiatives may also be dependent on the biased interests of CSOs. In the sample, top-down was most common, accounting for 66% of cases. In 19% of cases, CSOs or academics organized the CA bottom up. In 15% of cases, the CAs were organized hybrid.

**Figure 5.**
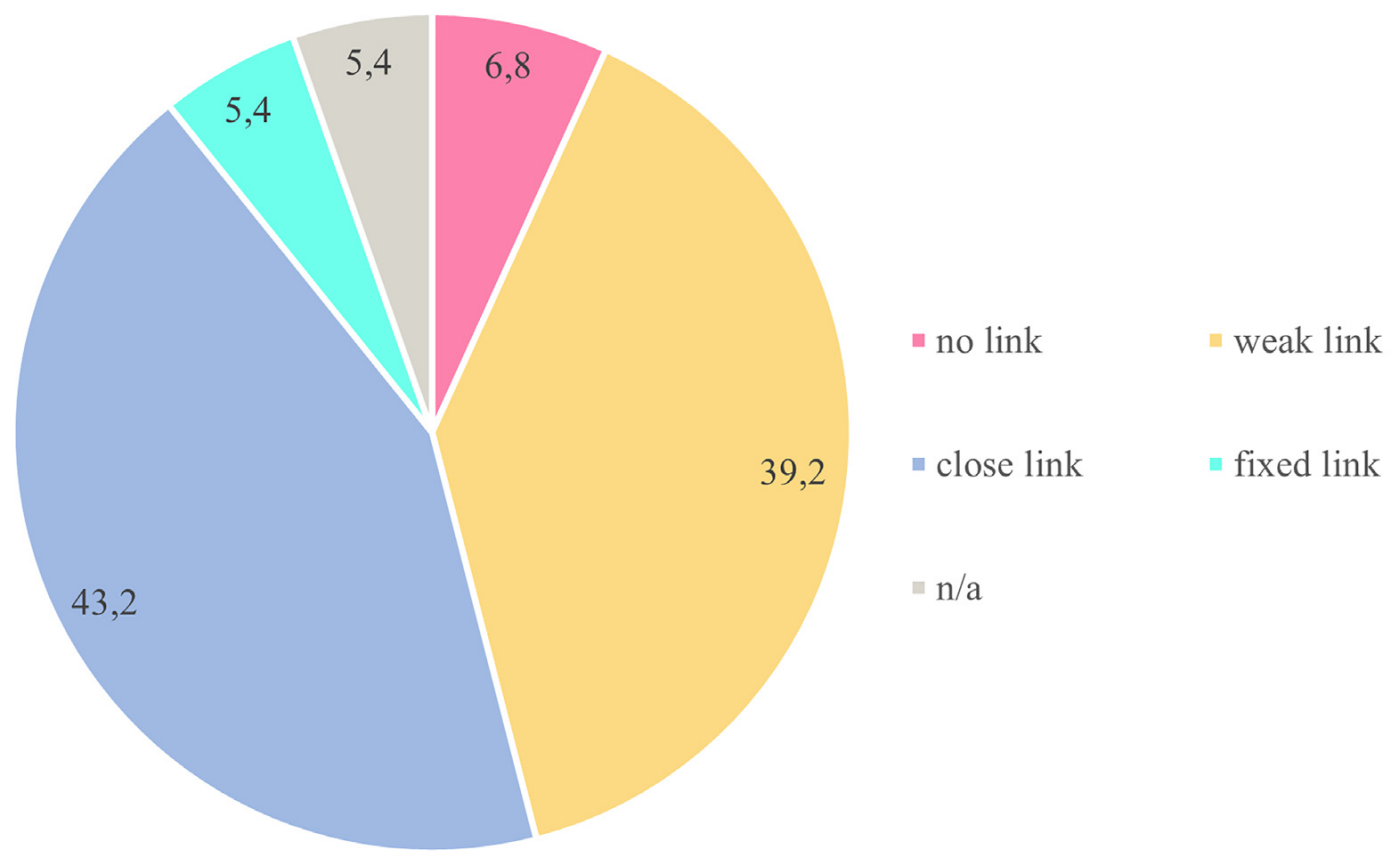
Type of connection between CA and political system in terms of uptake of CA recommendations. Source: Stack *et al.* (2023).

How did the CAs liaise with the responsible authorities *after* the CA regarding their recommendations, and how were they employed in policymaking? This connection between CA and the political system can be categorized as *“non-existent”*, *“weak”*, *“close”* or “ *fixed”*. Figure 5 shows distribution across cases.

In 39.2% of cases, the connection between CA and policymaking was weak. CA's recommendations were non-binding, and politicians declared that they might consider them once they received them. In 43.2% of cases, the responsible authorities accepted accountability for implementation and undertook to respond to them, reporting on whether and to what extent they had executed the CA recommendations received. If they decided not to implement specific recommendations, they provided an explanation. In some cases, the authorities regularly monitored the implementation of the recommendations received from a CA. This close connection between CA and policymaking was sometimes planned from the outset and sometimes decided during the process. In the Austrian Klimarat ( Buzogány *et al*., 2022), Berlin ( berlin.de, n.d.a), Bonn ( beteiligung.bonn4future.de, a; beteiligung.bonn4future.de, b), Mannheim ( mannheim-gemeinsam-gestalten.de, n.d.), Germany ( Bürgerrat Klima, 2021), Zealand ( Tekno.dk n.d.) and Turku ( Grönlund *et al*., 2022), the close link between CA and policymaking took the form of politicians participating in certain phases of the CA. In 5.4% of cases, e.g., in Mallorca ( Assemblea Ciutadana Pel Clima Mallorca, 2023), Gdansk ( Jestem z Gdanksa, 2016), Warzawa ( Eko.um.warszawa.pl, n.d. a; Eko.um.warszawa.pl, n.d. b; Eko.um.warszawa.pl, n.d. c) and Krakow ( Climate-kic.org, n.d.; Krakow.pl, n.d.; Smartcity-atelier.eu, n.d.) the authorities established a fixed connection, whereby politicians promised to consider recommendations as binding if they received sufficient support within the CA. In 6.8% of cases, there was no connection between the CA recommendations and the responsible authorities, either because the CA was experimental such as the Austrian “Zukunftsrat Verkehr” ( Frankus *et al*., 2023) and in Athens ( Arsinoe-project.eu, n.d.) and lacked an official mandate.

What did mapping reveal about the relationship between CAs and the political system? One aspect of this question relates to how politics and administration position themselves in relation to citizens, citizen engagement, and accountability. Do they consider them part of their self-perception? Andrews *et al*. (2022) highlight that the Scottish Parliament emphasizes citizen participation as a defining feature of Scottish identity to delineate itself from the Westminster model of policymaking. Andrews *et al*. (2022, p. 10) perceive the Scottish government as being particularly committed to “constant dialogue with Scotland’s people, listening, engaging and responding, and building on the principle that everyone is entitled to have the opportunity to shape Scotland’s shared future”. Grönlund *et al*. (2022) state in their report on the CA in Turku, that politicians participated in certain phases of the CA. This contributed to mutual understanding: citizens learned about local politics, while politicians learned about citizens' concerns and everyday life. Documents providing information about Dutch CAs in Amsterdam ( Postma *et al*., 2022; Teamburgerberaad.nl, n.d.), Den Haag ( Den Haag, 2023a; Den Haag, 2023b; Den Haag, 2023c), Zwolle ( Zwolle, n.d.a; Zwolle, n.d.b; Zwolle, n.d.c) indicate an exchange between policymakers and the CA. In these cases, a sequence of interactions existed between citizens and policymakers, with the CA making recommendations and the government responding, as well as monitoring activities. Such situations of accountability also occurred in Swiss Yverdon-le-Bains ( Yverdon-les-Bains, n.d.a; Yverdon-les-Bains, n.d.b; Yverdon-les-Bains, n.d.c) Brussels ( Assembleeclimat.brussels, n.d.; Buergerrat.de, n.d.), Berlin ( Abgeordnetenhaus von Berlin, 2022; berlin.de, n.d. a; berlin.de, n.d. b), Rouen ( Rouen citoyenne, 2022a; Rouen citoyenne, 2022b; Rouen.fr, n.d.), Lisbon ( Linde, 2023; Lisboa Camera Municipal, 2022a; Lisboa Camera Municipal, 2022b) and the UK ( Climate Assembly UK, n.d.). This contrasts sharply with CAs, such as those on energy poverty in Poland ( Fundacja Stocznia, 2022a; Fundacja Stocznia, 2022b; Fundacja Stocznia, 2022c) and transport and air pollution in Serbia ( Fiket & Đorđević, 2022), in which politicians showed no interest in citizen participation whatsoever.

Having shared political support is essential for a CA to be embedded in the political system. Buzogány *et al*. (2022), for instance, report that the Austrian Klimarat lacked backing from all parliamentary parties. Within the government, the CA was a prestigious project for one of the partners, which the other partner criticized. The climate ministry, which was headed by a Green Party politician, organized the CA with little intergovernmental coordination. The CA's close political connection to a single party and minister hindered its impact. Even CA participants were doubtful about the potential political impact of their recommendations. In a survey, 41% of members who participated in the final session said that they “had little or no confidence that policymakers would make an effort to implement the CA's recommendations” ( Buzogány *et al*., 2022, p. 14 ff.).

Another aspect of the political embeddedness of the CA concerns how citizens perceive their role in policymaking. In their report on two CAs, Fiket and Đorđević (2022, p. 2) portray Serbian citizens as “passive and apathetic, distrustful of democratic institutions and political representatives, and disappointed by the difficulty of influencing political decisions”. However, lack of trust in politics and institutions is not only a problem of a few countries; it is a serious global threat to democracy ( OECD, 2021; OECD, 2025). In Lisbon, for instance, a lack of trust in the political system, particularly among young people, was cited as a key motivation for establishing a CA ( Lisboa Camera Municipal, 2022a). Buzogány *et al*. (2022) report that a lack of transparency in the selection of CA participants conducted by the official statistical office created public mistrust in the Austrian CA. It was only after some public discussion that the recruitment methodology was made transparent.

### Stakeholder workshops on drivers and barriers of CAs

**Figure 6.**
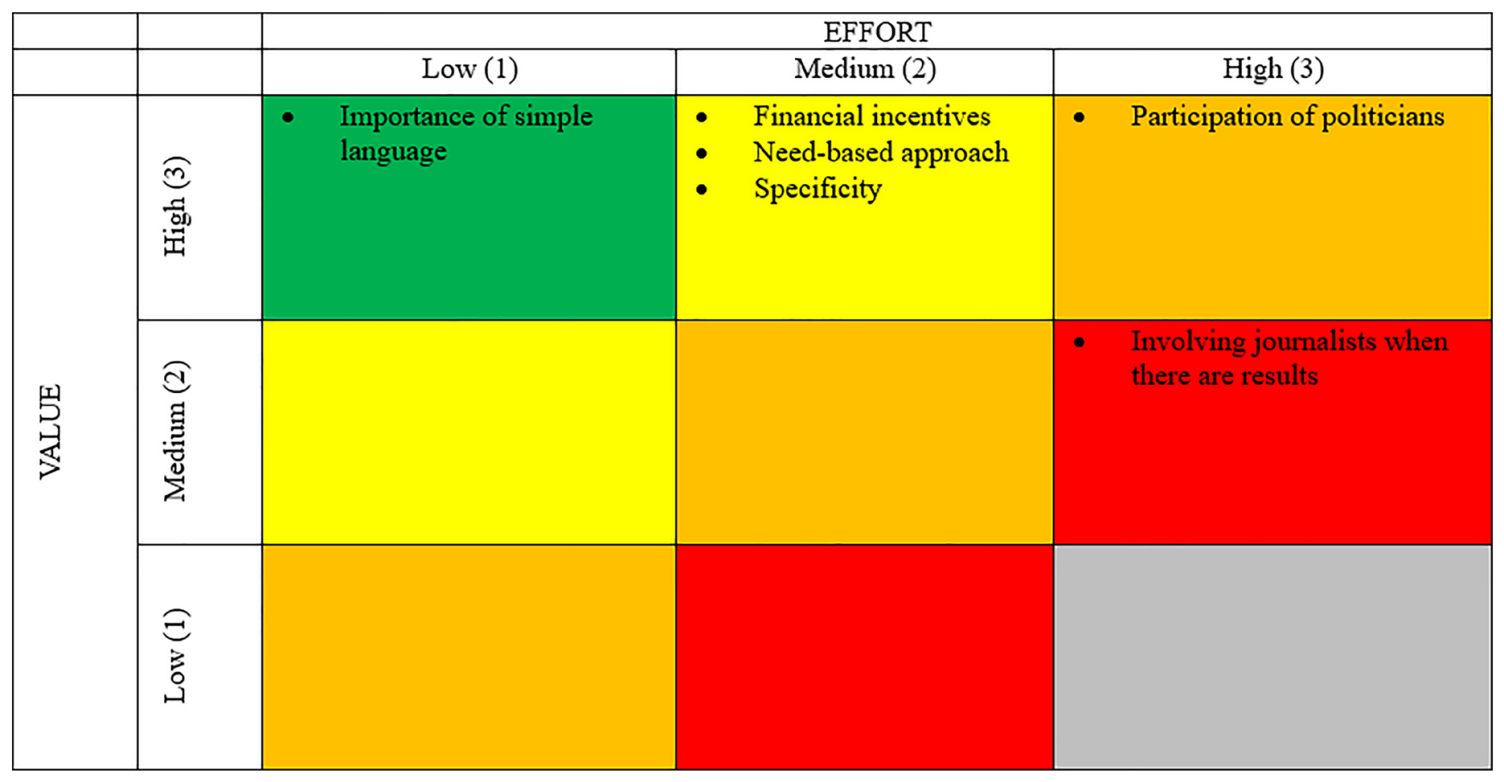
Drivers Priority Matrix. Source: Di Ciommo *et al.* (2023).

In workshops, stakeholders identified and ranked barriers and drivers of CAs on a three-point scale (high, medium, or low), according to their perceived relevance and the effort required to overcome them. Figure 6 shows the drivers for CAs, as identified by workshop participants, categorized according to their value and the effort required to achieve them.

The key drivers of successful CAs integration into policymaking were mostly considered to be of high importance. However, the effort required to address them varied. Important drivers identified in the workshops included measures to safeguard equality and inclusiveness in access, the relevance of the CA to citizens, and its impact.

### Drivers

***Importance of simple language.*** Participants agreed on the importance of using simple language throughout a CA, especially when engaging with a diverse group of people who have different levels of education and expertise. This makes information accessible to a wider and diverse audience. It facilitates understanding, enabling participants to engage more effectively in discussions and decision-making processes. It promotes inclusivity and ensures that the CA represents a diverse range of views.

***Offering financial incentives.*** Stakeholders shared different experiences from different EU countries and expressed diverging opinions on financial compensation for participants. Some said that compensation has no effect on participation, as people see it more as a civic duty. Others indicated that financial compensation could reduce intrinsic motivation to participate. However, some thought that citizens are usually very interested in participating when financial compensation is offered. Overall, there was consensus that financial compensation is more important for people of younger age and/or lower income. Some stakeholders suggested offering participants the option to donate the money they receive.

***A need-based approach helps to motivate citizens’ participation.*** An approach based on the needs of CA participants considers their different motivations, concerns, and requirements. This results in more personalized and meaningful engagement. It allows organizers to provide tailored incentives and support based on participants' specific needs and circumstances. When CA participants see that their individual needs are being met, they are more likely to actively participate in the CA. A need-based approach recognizes and responds to the unique needs and challenges of communities, thereby empowering them. This recognition fosters a sense of ownership and participation in the decision-making process. Based on the views expressed by stakeholders, a need-based approach may be advisable to motivate CA participation.

***Detailed topics instead of general and superficial ones.*** Detailed topics provide a clear and precise focus for deliberations. It enables participants to gain a deeper understanding of specific issues and facilitates more informed discussion. Specific topics enable in-depth analysis and exploration of the complexities surrounding climate-related challenges. This is key for developing well-informed and nuanced recommendations. As climate challenges vary across regions, detailed topics enable the consideration of local contexts and specific impacts.

***Politicians need to be part of the assembly design phase.*** Workshop participants stated that politicians should be involved in the design phase of the CA to ensure acceptance of the assembly's format and importance. Their involvement from the outset is crucial for the assembly's success, as political support is essential for implementing citizens' recommendations. Involving politicians from different parties in the design phase helps to ensure that the CA aligns with existing policy goals and strategies, and that it will remain valid in the event of political change. This fosters a greater sense of ownership of the process, which can lead to more sustained commitment and support throughout the assembly's lifespan.

***Involvement of journalists.*** Journalists are usually invited to attend the CA towards the end, to write about the results rather than reporting on the process itself. However, some participants recognized the value of engaging them during the assembly. Nevertheless, concerns were raised about the time required to participate for several days and then produce a brief report. Furthermore, some experts pointed out that the mindset of journalists can have a significant positive or negative impact (e.g., increasing awareness if they effectively communicate the importance of such events, or undermining credibility if they approach the CA with skepticism).

### Barriers and bottlenecks

**Figure 7.**
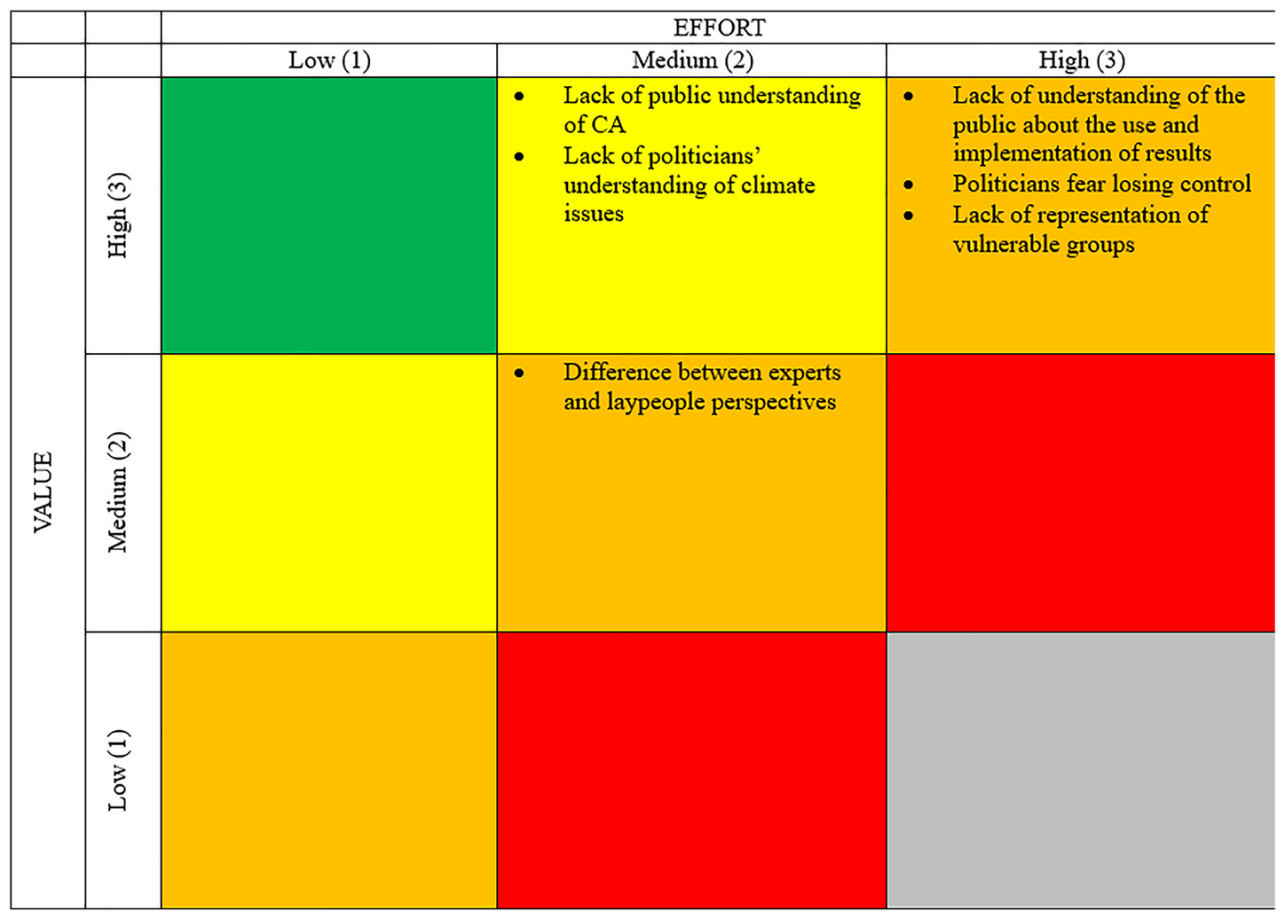
Barriers and Bottlenecks Priority Matrix. Source: Di Ciommo *et al.* (2023)

The barriers to CAs that were identified during the workshops included issues relating to knowledge, power and inclusiveness. While they were mostly assessed as having high value, overcoming them would require significant effort in many cases. Figure 7 depicts the barriers and bottlenecks for CAs, as identified by workshop participants, categorized according to their value and the effort required to overcome them.

***Lack of understanding what a CA is.*** Workshop participants thought that citizens might be reluctant to participate if they were unfamiliar with the concept of the CA or how it operates. This could limit representation and diversity in the CA, potentially excluding valuable perspectives. At the same time, the questions that frame a CA are often unclear, or are selected without due consideration of what a CA can achieve and how it operates. For instance, some experts noted that facilitators lack the scientific knowledge necessary to formulate appropriate questions, and that climate experts are not familiar with facilitating assemblies. Bringing these two groups together is a key component of designing a CA, which helps ensure the inclusion of relevant questions. Stakeholders also emphasized the importance of learning from past CAs and of empowering people rather than merely consulting them.

***Politicians are not always in a position to understand the need for climate actions.*** Workshop participants believed that politicians often face pressure to deliver short-term results, particularly when election cycles are short. However, climate change requires long-term thinking and sustained efforts, making it challenging for some politicians to prioritize it over issues with more immediate and visible impact. Some politicians may also lack a deep understanding of the scientific consensus on climate change. For instance, some workshop participants referred to local governments in rural areas that frequently neglect climate issues and lack a comprehensive grasp of sustainability. Providing education for everyone, including government officials, in rural areas would be beneficial. In other cases, the main problems are a lack of political commitment and insufficient resources. The biggest barrier is the urgent need to address climate change. Therefore, it was considered crucial to define which parts of climate policies and strategies participants should be involved in, given that their participation is expected to be valuable. Some parts (the most urgent) could be decided and implemented by governments, while citizens' participation would be useful for others.

***Lack of understanding of how precisely the results are going to be used and implemented.*** Stakeholders emphasized that a lack of understanding of how CA results will be implemented could significantly impact the success and legitimacy of the process. If the public is unaware of how CA outcomes will be used, this can lead to skepticism and distrust. A lack of clarity regarding the implementation of results may result in lower participation rates. If the public believes that recommendations are unlikely to be implemented, this can undermine the CA’s overall credibility. Stakeholders, including citizens, may be less willing to actively collaborate in the CA if they are uncertain about how the results will be used and implemented. One possible approach to addressing the issue of politicians failing to implement CA proposals is to co-create solutions with them by involving them in the CA process. This contrasts with citizens creating solutions and proposing them to politicians for implementation.

***Politicians are afraid of losing control.*** Stakeholders thought that politicians might be concerned that implementing recommendations from a CA could constrain their options and limit their autonomy. This fear may be particularly pronounced if recommendations challenge established political interests or traditional policy directions. CA participants often represent a variety of viewpoints, and their recommendations may include politically challenging or unpopular measures. Politicians may therefore fear electoral consequences or backlash if they endorse such recommendations. Climate change is a complex and interconnected challenge that requires multifaceted solutions. They may be apprehensive about endorsing recommendations that require coordinated action across various sectors and levels of government, as well as sustained effort over an extended period. The main obstacle is that politicians must empower citizens and consider the consequences. When politicians do not use or support the CA's recommendations, it is often because they have a fear of losing power. Creating a collaborative and supportive environment between politicians and CA participants is essential to overcoming these fears and ensuring the recommendations result in meaningful and effective climate policies. Ultimately, politicians need to be involved in order to learn how to approach politics differently.

***Lack of inclusion and representation because participants tend to be highly educated.*** CAs, which aim to gather diverse perspectives, can encounter problems relating to the socioeconomic, educational and demographic characteristics of participants. Those with higher education levels or greater awareness of environmental issues may be more likely to volunteer for or participate in such assemblies, creating a self-selection bias. Participation in CAs may require a time commitment and incur costs, which could act as barriers for individuals with lower socioeconomic status or who face practical challenges, such as childcare or transport issues. Additionally, outreach efforts may not effectively reach or resonate with diverse communities, potentially leading certain groups to feel excluded or unaware of the opportunity to participate.

***Experts versus citizens.*** While the input of experts is valuable for providing specialised knowledge, it is also essential to ensure a comprehensive and inclusive deliberative process. This can be achieved by seeking perspectives from non-expert organizations and associations, particularly those representing diverse interests and communities. These organizations often represent specific communities, industries, or interest groups. Involving these organizations in decision-making processes ensures that the concerns and interests of their stakeholders are considered, thereby promoting inclusivity and democratizing decision-making.

### Survey of cities and regions on the practice of citizen participation

**Figure 8.**
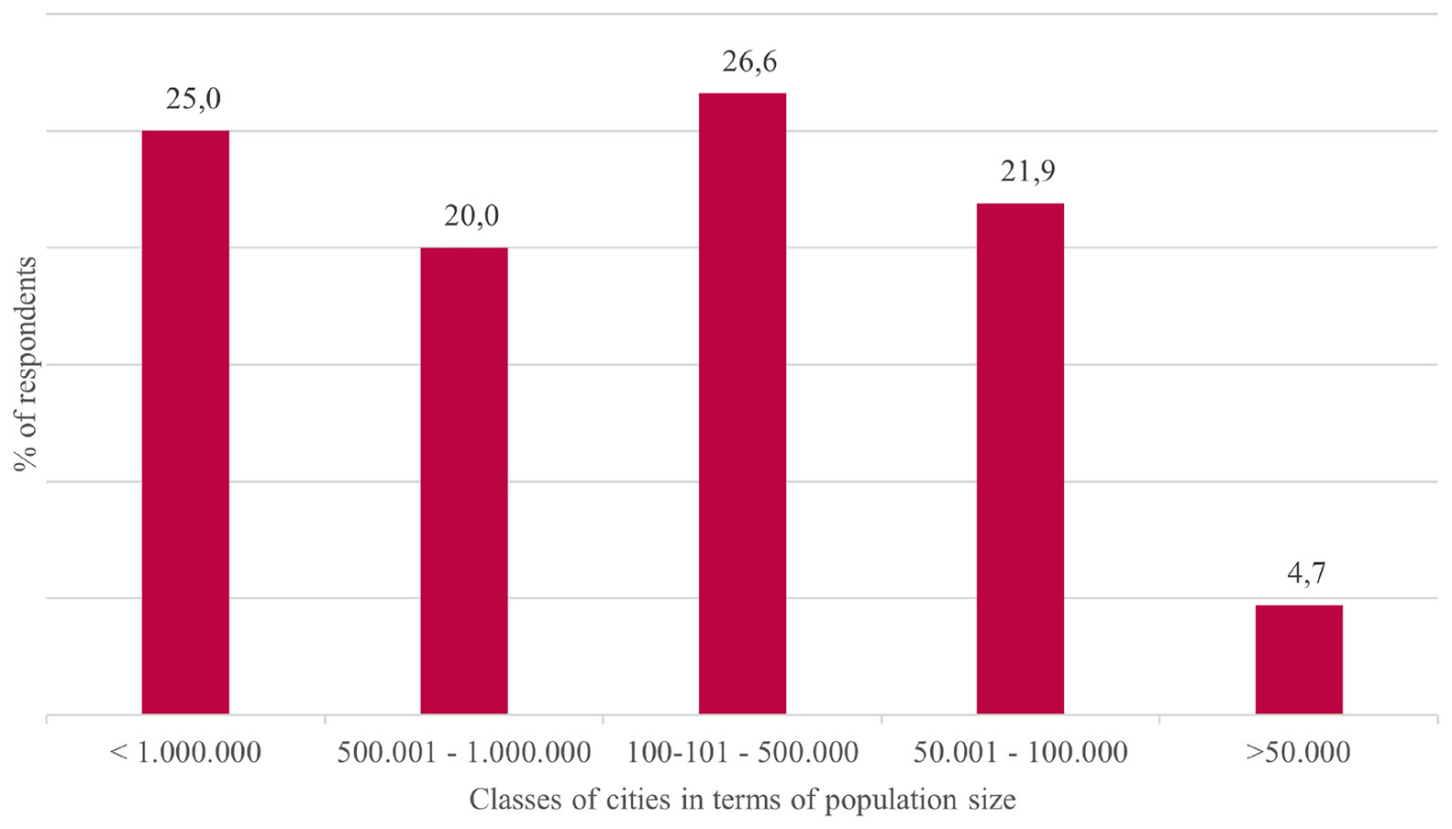
Share of responding cities/regions by size of population in percentage. Source: Cristea *et al.* (2023)

Sixty-four European cities and regions responded to the survey, with 75% representing cities and 25% representing regions. Respondents were unevenly distributed in terms of population size ( Figure 8).

### Citizen engagement in climate policy

The survey shows that climate policies have become a well-established practice in many European cities and regions. 82.8% of the responding cities and regions had explicit climate change policies or related strategies. 6.3% stated that climate policies were cross-cutting issues incorporated into other policies and strategies. 10.9% said that their region or city had no climate change policy.

According to the survey, European cities and regions use various engagement approaches to develop climate change policies. The most prevalent approach is to involve city officials, experts, stakeholders and citizens (50%), followed by the development of policies with the input of experts only (31.3%). An expert-based approach excludes citizens and civil society, indicating a lack of participatory governance. 10.9% of respondents said public authorities’ officials developed policies in-house, using internal institutional capacity. These processes may show administrative self-reliance, but they lack stakeholder oversight, raising concerns about transparency, inclusiveness and accountability. Only 9.4% of respondents said that city officials developed climate change policies with input from stakeholders, including citizens.

Despite some public engagement in climate policy, the survey shows that citizen participation is infrequent. Only 15.6% of respondents said it happened regularly, at least once a month. 34.4% said their city or region would engage with stakeholders, including citizens, occasionally in the development of climate change policies, typically once every few months. Another 28.1% said this would happen rarely, typically once or twice a year.

**Figure 9.**
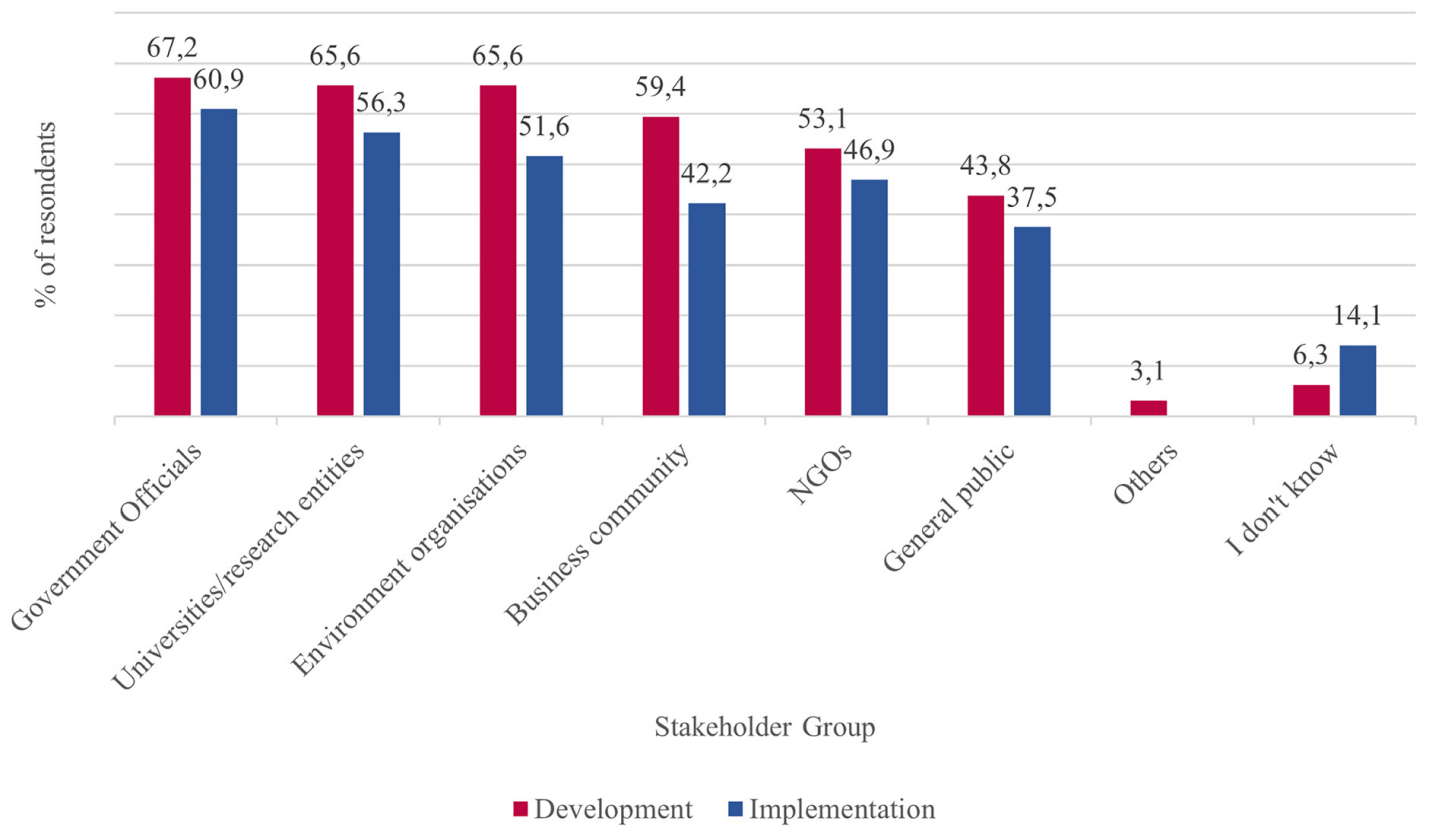
Stakeholders involved in developing and implementing climate change policies and measures in percentage of respondents (multiple answers possible). Source: Cristea *et al.* (2023)

In addition, the involvement of stakeholders in policy development and implementation varies according to the groups of actors, with some stakeholder groups being more heavily represented than others. Government officials, university and research entities, environmental organisations, the business community, and NGOs were more often included than the general public. Furthermore, the level of involvement for all actors is higher during policy development than during policy implementation (see Figure 9).

The survey of European cities and regions also revealed that, while some of the results of stakeholder and citizen engagement activities were adopted, results were not always translated into policies. 48.4% of the respondents reported that output was integrated into plans and strategies for achieving goals and targets. 37.5% used the output to identify and prioritize policy actions and measures. 34.4% used it to inform policy development and decision making and 29.7% incorporated it into policy goals and targets. 29.7% did not know how to answer the question.

Cities already use various on- and offline methods to engage with stakeholders, including citizens. Community events and meetings that allow direct interaction and dialogue with the community are widely used for stakeholder engagement (68.8%). Social media (53.1%), that enables real-time communication and interaction with a diverse audience, is the second most used platform for stakeholder engagement. The use of collaborative partnerships with an array of organizations and entities is emphasized by 50.0% of responses, while 45.3% mentioned the use of online surveys for stakeholder engagement. This approach provides a practical and flexible option for collecting input and feedback from a broad audience. 39,1% use educational programs and workshops as a method to inform and involve stakeholders. Citizen assemblies and participatory budgeting are less frequently used methods of public engagement, with 34,4% and 31,3%, respectively.

Social media and online platforms (73.4%), public meetings and forums (62.5%) as well as traditional media (59.4%) were communication channels cities/regions used most often to communicate with stakeholders/citizens. One-on-one meetings that allow for in-depth discussions and tailored communication was used by 42.2% of the respondents, while workshops and training sessions with the same percentage highlight a commitment to more interactive and educational forms of communication. The least common channels are newsletters, with 39.1%.

The survey showed that CAs are the exception in European cities and regions. Only 23.8% of responding cities and regions had organized them. 55.6% did not organize a CA, and 20.6% of respondents were unsure. If CA were carried out, local and regional authorities play a vital role in organization. CA were either organized by local (51.7%) or regional authorities (18.8%). Civil society organized CA less frequently. 6.5% of the CA were organized by NGOs and 6.8% by private entities. In 11.7% of the cases, respondents did not know the organizers, in 4.7% the organizers were other entities.

The survey also revealed that cities and regional administrations find it challenging to engage with the stakeholders on climate change issues. 50.0% of respondents identified reaching and engaging with diverse groups of stakeholders and end users as a main challenge when engaging with stakeholders (including citizens) on climate change issues. Other challenges included limited resources or funding to support engagement efforts (46.9%), and a lack of interest or awareness of climate change issues among stakeholders and end users (45.3%). 4.6% of respondents cited resistance to change or opposition to climate change policies as an issue. Interestingly, a significant proportion of respondents (37.5%) perceived participation fatigue as an issue. While 28.1% mentioned inadequate instruments or procedures as the main challenge, only 3.1% said that there were no challenges.

When asked about the main challenges involved in engaging stakeholders (including citizens) in the implementation of climate change policies, the most frequently cited one was aligning stakeholder and end-user priorities with policy objectives (46.9%). This was followed by limited capacity (43.8%), lack of resources or funding to support engagement efforts (42.2%), resistance to change or opposition to climate change policies (37.5%), and lack of expertise among stakeholders and end users (29.7%).

## Discussion

### Climate Assemblies: A wave that is more of an exception than the rule

The survey shows that climate policies have arrived in European communities and regions. Almost 83% of responding cities/regions said they had a climate change policy or related strategy. However, citizens have yet to become involved in developing and implementing these policies. Although mapping CAs in line with OECD data ( OECD, 2021) shows a remarkable increase in CAs in European countries and regions, survey data indicates that CAs are the exception rather than the rule. Although the vast majority of responding cities and regions (81.2%) stated that they had engaged with stakeholders (including citizens) when developing climate change policies, this engagement was rather infrequent. 15.6% of respondents said it happened regularly, 34.4% occasionally and 28.1% rarely. Furthermore, different types of stakeholders have unequal opportunities of getting involved in policy development, with government officials (67.2%), researchers (65.6%), environmental NGOs (65.6%) and businesses (59.6%) being the most favored. In comparison, the general public (43.8%) stands a lower chance of being engaged. Furthermore, as policies progress from the development stage to the implementation stage, it becomes less likely that stakeholders from outside government are involved. In addition, the outcome of stakeholder engagement is often not incorporated into the development of climate change policies.

### Political commitment and link to policy making/impacts on policies

The key to successful implementation of CA recommendations is embedding the process within political and administrative systems. The connection between CA and the established political system is crucial for translating CA recommendations in actual policies. The mapping exercise revealed that this link varied significantly across Europe in both fashion and strength, ranging from a “fixed link” (5.4%) to a “close link” (43.2%), to a “weak link” (39.2%), to “no link at all” (6.8%). A “fixed link” makes CA recommendations binding for policymakers if they reach a certain support threshold from the CA. With a “close link”, policymakers are accountable for how they deal with recommendations or their inclusion during the CA process. With a “loose link”, policymakers simply receive CA recommendations with no defined follow-up process. A “no-link” scenario means that policymakers refuse to receive policy recommendations or to participate in CAs. Several of the barriers and bottlenecks identified by stakeholders and experts in workshops concern the connection to and integration with the political system, on the part of both citizens and policymakers. Experts and stakeholders mentioned that citizens might be skeptical of CA because they are unsure about the implementation of results, in other words, the binding nature of CA recommendations and the accountability of policymakers. They also mentioned that politicians might fear losing control because of CAs.

### Political culture matters

The link between CA and policymaking is not arbitrary but is closely connected to the political and administrative context in which the CA is developed and embedded. Such a context can encourage or discourage citizens from participating in politics and administration. Fiket and Đorđević (2022) use the concept of “political opportunity structure” to explain the challenges faced by grassroots CAs in Serbia. They emphasize that the political environment affects citizen mobilization and social movements, as well as their ability to mobilize and achieve collective goals, through “constraints, possibilities and threats” ( Fiket & Đorđević, 2022, p. 2).

The concept of “political culture” ( Hanisch, 2005) adds historical depth to the notion of political context. It helps to understand whether and how participation is possible within a given political system, considering its history and the respective configuration of actors. *Political culture encompasses “a set of orientations, attitudes, and opinions regarding political processes and structures; but also, behavioral patterns in the sense of political mentality research, which are learned through historical traditions and supported by political symbols”* ( Hanisch, 2005, p. 23ff. translations the authors). Political culture encompasses the entire "population (national culture), the culture of large groups (camp culture) and of functional elites (elite culture). Political culture is associated with “deep structures of values” and the “long duration” of a seemingly “immutable history.” Importantly, it includes “mostly unreflective, even unconscious attitudes, basic mental processes, and everyday references” ( Hanisch, 2005, p. 23ff.). National history, self-understanding and role of actors (e.g., officials and politicians) and institutions (e.g., parliaments, governments, heads of state, associations) within a given political system, the perception of the legitimacy and place of citizen participation within that system, as well as long, -medium and short-term historical experiences with it, play an important role for the implementation of participatory events such as CAs.

The mapping exercise revealed differences in political culture that affect the development of CAs and the implementation of their results. While CAs in Germany, the Netherlands, Switzerland, Ireland and Scotland were characterized by collaboration between politicians and the CA, cases in Poland and Serbia saw politicians who refused to collaborate. In two Austrian cases, for instance, the connection between CA and policymaking was either non-existent ( Frankus *et al*., 2023) or relatively weak ( Buzogány *et al*., 2022; klimarat.org, n.d.), however, it was stronger in a third Austrian case in a province with a long tradition of civic participation ( OECD, 2021; Vorarlberg.at, n.d.; Vorarlberger Landesregierung, 2021; Vorarlberger Landesregierung, n.d.). In Switzerland, a country with a strong tradition of direct democracy, a parliamentary motion to establish a permanent and powerful CA with a direct link to policymaking was rejected because its opponents believed that it would compete with existing representative and direct democratic procedures ( Die Bundesversammlung, 2020; Die Bundesversammlung, 2021; Nationalrat, 2021). These examples demonstrate national and even regional differences in Europe in how politicians perceive their role in democracy and policymaking and the role of citizens in policymaking outside of traditional elections. They also demonstrate variation across Europe in policymakers' acceptance of accountability for CA recommendations. The question to what extent politicians are ready to accept recommendations of CAs is connected to power. In line with this argument, stakeholders mentioned during workshops that politicians have a fear of losing control, which is an important barrier to CAs that is difficult to overcome.

However, political culture also encompasses how citizens perceive their own role in politics and how much they trust politicians and political institutions to implement recommendations from CAs. During the online workshops with stakeholders and practitioners of CAs, translating CA results into politics was identified as an important, yet difficult-to-overcome barrier. The survey on citizen participation in cities and regions also showed that citizen engagement in cities and regions often does not always translate into concrete policies. In line with this, participants in the Austrian Klimarat expressed skepticism about whether the results would actually be implemented ( Buzogány *et al*., 2022). Fiket and Đorđević (2022) report that in Serbia, the “sense of political efficacy” — the belief that political activities make a difference — is low among citizens and that participation in parliamentary elections has decreased over the last two decades, with only a minority engaging in non-institutional political participation. Conversely, some forms of non-institutional political protest have increased in recent years.

## Conclusion

The escalating challenges posed by climate change are not only environmental crises but also crises of governance and representation. As climate emergencies unfold, communities and their citizens are not passive bystanders, but vital stakeholders with a responsibility and right to be represented in the measures devised in response to these crises. However, the current political apparatus has not been designed with this sense of urgency and coordinated action in mind. Against this backdrop, CAs have emerged as an innovation in democratic engagement. They provide a platform for civil discourse and collective decision-making, intervening directly in the typical hierarchical and technocratic approaches to climate policymaking. Drawing on diverse community representation, they facilitate deliberation on feasible climate adaptation measures. The strength of CAs lies in their ability to foster legitimacy, accountability and social cohesion around difficult decisions ( OECD, 2021). As an innovation in representative democracy, CAs nevertheless challenge politicians, public administrators, and citizens to reconsider their self-perception, roles, and practices within the current political system. Nevertheless, all too often, the placement of CAs within their social and political context fails to leverage their potential for democratic revival in favor of procedural purity. Specifically, critical factors such as coalition building and raising awareness are underrepresented in the discourse surrounding climate assemblies, as evidenced by media reports and evaluation studies.

The paper has shown that there is ample experience of CAs in Europe that face certain drivers and barriers. Climate policies are well represented in European cities and regional policy agendas, yet there is little experience in institutionalized two-way citizen engagement on national, regional and local levels. The phrase "we'll cross that bridge when we get there" encapsulates the prevalent narrative of the reactive nature of implementing CAs. Before navigating the multifaceted challenges of organizing CAs, addressing the needs of assembly members and managing interactions with experts, stakeholders must first persuade political leaders to accept the concept. An analysis of existing frameworks reveals a tendency to focus predominantly on process adaptations rather than the social strategies underlying their success. Without such strategic approaches, the potential of CAs is limited to political leaders who accept them as temporary exercises, sometimes reluctantly, rather than as meaningful opportunities to integrate innovative participation into decision-making processes. Yet, CAs and similar participatory approaches should be designed not just to produce recommendations, but also to transform climate policy by embedding participation in its fabric via firm institutionalization ( KNOCA, 2022b; OECD, 2021).

In order to ensure that the function of CAs is not merely symbolic, but that they have a tangible impact on the policymaking process, it is crucial to establish institutional frameworks that facilitate the provision of feedback and the subsequent implementation of citizens' recommendations. These frameworks determine how citizens' recommendations are reviewed, responded to, and potentially implemented by public authorities. Without such structures, even well-conducted deliberative processes risk being ignored or sidelined. A clear transparent institutional pathway is necessary to demonstrate that citizens' input is valued and can lead to real change. This strengthens democratic legitimacy and public trust in the CAs and public institutions. Ultimately, institutional frameworks facilitate the integration of CAs as innovative forms of participation in decision-making processes.

A strong institutional framework starts with a formal mandate. For example, establishing a CA under the auspices of a public authority — such as a national or regional parliament, or a ministry responsible for climate policy — ensures that the process is embedded in the institutional decision-making environment. Ideally, this mandate should include a commitment to respond to the CA's findings within a specified timeframe, explaining how recommendations will be integrated, modified or rejected. This 'obligation to respond' mechanism is a cornerstone of institutional accountability.

The survey shows that the role of CAs must be strengthened in local and regional climate governance frameworks. While expert consultations and public meetings are used to engage stakeholders, CAs offer a unique opportunity to institutionalize deliberative democracy and ensure that citizen voices are heard and meaningfully integrated into decision-making processes. The results show that many public authorities still use top-down or expert-driven approaches with little stakeholder engagement, especially from citizens. Engagement mechanisms frequently struggle to translate into policy development or implementation. Thus, enabling the role of CAs and integrating them with other engagement instruments will help create a stronger, democratic and socially cohesive approach to climate adaptation and mitigation.

## Key policy insights

- Citizen assemblies (CAs), a form of deliberative democracy, have increasingly been used in climate policies. In order to be successful democratic innovations, CAs must be inclusive, visible and meaningful to citizens, and their results must have an actual impact on policymaking.
- The roles, self-perception and responsibilities of policymakers, administrators and citizens differ across Europe and are distributed differently between these actors within a specific political culture. These political cultures have evolved from country-specific long-term historical and political experiences, providing the context in which a CA operates. It is therefore necessary to identify elements of a political culture that hinder deliberative democracy and consider what actions are necessary to strengthen the structures, roles and practices that promote deliberative democracy within a political culture.
- In order to ensure its impact, legitimacy, transparency, and accountability the commissioner of a CA must ensure that the coupling of the event with policymaking is appropriate to the specific cultural, social, political and environmental context.

## Ethics and consent

Ethical approval was not required.

Verbal informed consent was obtained during the online workshop, as participants had been previously informed—prior to recording—about the anonymized use of any information shared during the session. A free, prior, and informed consent (FPIC) approach was employed to ensure that all participants were fully aware of the nature, purpose, and implications of their participation before providing their consent. Participants of the online survey were informed that all personal data is collected only upon receiving their informed consent for data collection, processing, and usage, and any participant providing personal data can withdraw their participation and related data from the survey or interview at any time. An informed consent form was provided. No ethical approval for workshop and survey were obtained because no special categories of personal data or personal data related to criminal convictions and offences was collected.

## Data Availability

IRIHS repository: CLIMAS-EU survey https://doi.org/10.60739/IHS-7296 ( Andrei & Cristea, 2023) The project contains the following Underlying data: andrei-cristea-2023-climas-eu-survey-data.xlsx IRIHS repository: CLIMAS-EU survey https://doi.org/10.60739/IHS-7296 ( Andrei & Cristea, 2023) The project contains the following Extended data: Topic guide City_Region interview Informed consent slide CLIMAS workshops Informed consent slide CLIMAS online survey CLIMAS Survey T2_3 Data are available under the terms of the Creative Commons Attribution 4.0 International license (CC-BY 4.0).
