# Supplementary material for: Citizen participation in climate politics. Drivers and barriers of Climate Assemblies in Europe
Source: Open Res Eur. 2026 Jan 2;6:3. [Version 1] doi: 10.12688/openreseurope.21452.1 (PMC12873538; doi:10.12688/openreseurope.21452.1)
Supplement: Peer review history

# Reviewer report: Reviewer response for version 1

**DOI:** 10.21956/openreseurope.23203.r68014

**Published:** 2026-02-17

**Authors:** Subhashree Nath

**Affiliations:** 1 Leibniz Institute of Ecological Urban and Regional Development, Dresden, Germany

**General:**

 + Figures which have been adapted from a source, should say so. For example, those adopted from Stack et al. 2023

 + Figure 3 should have a legend of the abbreviations

 + Some of the references don't have DOI/web link. For example, Stack et al 2023

**Introduction**

 + Mentions both CA as an innovation in democracy and later as a policy innovation. How are they different? How is it a policy innovation?

**Methods**

 The method is well thought and the inclusion of different approaches complement each other. However, in my opinion, the section would benefit from the following:

 + What is the reason for using this certain Priority Matrix that was adapted(?) from Di Ciommo et al? 

 + How was the degree of importance of various perceived drivers/barriers agreed on? Did you also keep track of how many votes each of the drivers/barriers receive? 

 + What questions were asked in the workshop?

**Results and Discussion is well written.**

If applicable, is the statistical analysis and its interpretation appropriate?

Yes

Is the study design appropriate and is the work technically sound?

Yes

Is the work clearly and accurately presented and does it engage with the current literature?

Yes

Are the conclusions drawn adequately supported by the results?

Yes

Are sufficient details of methods and analysis provided to allow replication by others?

Yes

Are all the source data and materials underlying the results available?

Yes

Reviewer Expertise:

Transformative community-based climate change adaptation; Urban Planning

I confirm that I have read this submission and believe that I have an appropriate level of expertise to confirm that it is of an acceptable scientific standard.

# Reviewer report: Reviewer response for version 1

**DOI:** 10.21956/openreseurope.23203.r67572

**Published:** 2026-02-04

**Authors:** Irena Fiket, Nemanja Andjelkovic

**Affiliations:** 1 University of Belgrade, Belgrade, Serbia; 2 Institute for Philosophy and Social Theory, University of Belgrade, Institute for Philosophy and Social Theory, Beograd, Serbia, Serbia

The paper, "Citizen participation in climate politics: Drivers and barriers of Climate Assemblies in Europe," represents a significant empirical contribution, based on very rich data, to the study of deliberative minipublics and their application to climate policymaking. We found the article very interesting, with a highly relevant topic and a solid methodological approach. However, we believe some important aspects need improvement. While the paper makes a meaningful empirical contribution in this regard, it would benefit from a more thorough review of previous relevant studies, as the research gap the authors aim to address is not clearly identified due to the underdeveloped discussion of literature pertinent to the research questions. 

 First, the manuscript lacks engagement with the literature on uptake of deliberative recommendations, which is particularly relevant for the third research question concerning policymakers’ readiness and ability to incorporate Climate Assemblies and their outputs into policy processes. This omission is significant, as this body of work offers conceptual tools for distinguishing different dimensions and degrees of uptake, as well as for operationalizing what “incorporation” of deliberative outputs actually entails. In this regard, the authors could benefit from consulting, among others, (Refer to reference no. 1) (Original work published 2020) and (Refer to reference no. 2)

 Without engaging with this literature, it remains unclear how the authors conceptualize and assess uptake, and how their findings advance existing debates.

 Second, the article would benefit from incorporating insights from deliberative systems theory, which could provide a more robust framework for framing and interpreting the third research question and the related empirical results. A systemic perspective would allow the authors to situate Climate Assemblies within broader deliberative and policymaking ecosystems. In this respect, the volume by (Refer to reference no. 3)

 Third, although the concepts of embedding and coupling are briefly mentioned in the text, they are not sufficiently elaborated in the theoretical framework, even though the empirical analysis relies on a typology of weak, close, and fixed embedding derived from these concepts. The authors should more clearly develop these concepts and situate them within the relevant literature on participatory governance, for instance, by engaging with (Refer to reference no. 4)

 Doing so would strengthen the analytical foundations of the typology used and clarify its theoretical implications.

 Furthermore, given that the authors discuss barriers, hurdles, and limitations related to both the deliberative process and policy uptake, the literature review would also benefit from engaging more explicitly with critical perspectives on deliberation and deliberative failures. Relevant contributions include: (Refer to reference no. 5), (Refer to reference no. 6), (Refer to reference no. 7), (Refer to reference no. 8) 

 Finally, while the literature review touches upon a wide range of topics, such as inclusion, recruitment, legitimacy, and political culture, the links to each research question are not always made explicit. A clearer conceptual mapping of constructs, mechanisms, and indicators for each research question would substantially improve the coherence of the theoretical framework. As it stands, the framework remains rather synthetic, combining elements of deliberation, coupling, and political culture, but without formal propositions or a clear measurement model. This results in some ambiguity regarding which elements of the framework are intended to address which research questions, and ultimately weakens the assessment of the paper’s contribution.

 At the same time, the breadth and ambition of the three research questions raise the issue of scope. It seems that these research questions may be too extensive to be fully and systematically addressed within a single article and might be more appropriately explored through a series of papers. 

 Nevertheless, the manuscript represents a significant contribution. By situating the study more clearly within the existing academic debate (a more focused and clearly articulated theoretical framework and a tighter alignment with research questions, concepts, and empirical analysis), the authors would enhance the paper’s contribution to the literature on the topic.

 Regarding methodology, a few issues merit further clarification.

 The qualitative analysis relies on inductively generated codes, yet the paper does not provide sufficient information on the coding protocol. In particular, it would be helpful to know how the coding process was organized, whether any form of intercoder reliability was applied, and how individual codes were ultimately aggregated into the four-category typology of institutional connection (“absent,” “weak,” “close,” “fixed”). Providing a rudimentary codebook, illustrative examples, or clearer decision rules for assigning connection types on a case-by-case basis would significantly strengthen methodological transparency. 

 In addition, stakeholder workshops, while highly valuable for identifying perceived drivers and barriers, are not without problems. The identified drivers and barriers primarily reflect stakeholder beliefs and experiences rather than directly observed outcomes. To reduce subjectivity, these findings could be further triangulated with documented process characteristics and observable outputs from the mapped cases (such as formal responses, policy uptake, or institutional follow-up mechanisms).

 Some conclusions drawn in the paper are clearly warranted by the data provided. For example, the claim that Climate Assemblies remain an exception in European cities and regions is well supported by the survey evidence and the descriptive trend mapping. Similarly, the argument that political support and institutional linkages shape the influence of Climate Assemblies is convincingly illustrated through multiple case examples.

 At the same time, there are instances where the conclusions tend to overreach the evidence. While the above claims are valid given the data, they should be framed more explicitly as exploratory, particularly in light of the limitations of the research design discussed earlier. Where possible, some conclusions could also be strengthened by moving from illustrative case-based evidence toward more explicitly comparative analysis, drawing on the already collected dataset. Finally, the transition from case to the broader claim that political culture matters for the effectiveness of Climate Assemblies is plausible but has not been systematically tested across a sufficiently large number of cases.

 Overall, the paper offers several important contributions. It provides a comparative and operational typology of the links between Climate Assemblies and policymaking, including their frequency across Europe; valuable empirical evidence on who commissions Climate Assemblies and why these matters for their impact; and a prioritized, practice-based account of key drivers and barriers to the meaningful use of Climate Assemblies. In addition, the governance level diagnosis showing that Climate Assemblies remain relatively rare and weakly institutionalized across Europe is particularly important. Taken together, these findings constitute a significant contribution to both the study of deliberative systems and applied climate governance.

 Precisely because of these strengths, the paper could further enhance its contribution by more explicitly engaging with deliberative systems theory, which offers a particularly suitable analytical lens for interpreting the comparative, governance-level insights generated by the study.

 In sum, the manuscript’s contribution lies in its empirical mapping of Climate Assembly policy embeddedness across Europe, its analysis of commissioning structures, its catalogue of practice-based drivers and barriers, and its governance-level diagnosis of the participation and policy-uptake gap. However, an open and recurring issue is that the theoretical framework remains rather synthetic, as we explained above.

If applicable, is the statistical analysis and its interpretation appropriate?

Partly

Is the study design appropriate and is the work technically sound?

Partly

Is the work clearly and accurately presented and does it engage with the current literature?

Partly

Are the conclusions drawn adequately supported by the results?

Yes

Are sufficient details of methods and analysis provided to allow replication by others?

Partly

Are all the source data and materials underlying the results available?

Partly

Reviewer Expertise:

Deliberative democracy, participation, and democratic innovations

We confirm that we have read this submission and believe that we have an appropriate level of expertise to confirm that it is of an acceptable scientific standard, however we have significant reservations, as outlined above.

## References

[1] : The Ideal of Uptake in Democratic Deliberation. *Political Studies* .2020;68(2) : 10.1177/0032321719858270 504-522 10.1177/0032321719858270

[2] : Deliberative Impacts: The Macro-Political Uptake of Mini-Publics. *Politics & Society* .2006;34(2) : 10.1177/0032329206288152 219-244 10.1177/0032329206288152

[3] Deliberative Systems.2012; 10.1017/CBO9781139178914 10.1017/CBO9781139178914

[4] : Introduction: Embedding participatory governance. *Critical Policy Studies* .2022;16(2) : 10.1080/19460171.2022.2053179 133-145 10.1080/19460171.2022.2053179

[5] : Deliberation, Participation, and Democratic Legitimacy: Should Deliberative Mini‐publics Shape Public Policy?. *Journal of Political Philosophy* .2015;23(1) : 10.1111/jopp.12031 40-63 10.1111/jopp.12031

[6] : The Lottocratic Mentality.2024; 10.1093/9780191982903.001.0001 10.1093/9780191982903.001.0001

[7] : The Derailed Promise of a Participatory Minipublic: The Citizens’ Assembly Bill in Flanders. *Journal of Deliberative Democracy* .2020;16(2) : 10.16997/jdd.402 10.16997/jdd.402

[8] : The limits of representativeness in citizens' assemblies: A critical analysis of democratic minipublics. *Journal of Sortition* .2025;1(1) : 10.53765/3050-0672.1.1.137 137-159 10.53765/3050-0672.1.1.137

# Reviewer report: Reviewer response for version 1

**DOI:** 10.21956/openreseurope.23203.r68009

**Published:** 2026-01-28

**Authors:** Diana Reckien

**Affiliations:** 1 University of Twente, Enschede, The Netherlands

**Review “Citizen participation in climate politics. Drivers and barriers of Climate Assemblies in Europe** **“**

 Section “ **Research Questions**“

 I would delete this phrase “Using a mixed method approach” at the beginning of the section on research questions, as you do not use this approach to raise the RQs.

 Section **Results**

 This section would benefit from revision. I find it logical to first report the survey results (i.e., "Survey data shows..."), reporting what is happening on the ground. Then, I feel, the information on CA fits in, as this relates more to how the local policies were developed (or not).

 Also, currently, it is not clear in what  way the “political culture, the specific roles of citizens, policymakers and administration therein” is significant. This sentence reads as generic. Can it be made more specific, e.g. by mentioning how it contributes to CA?

 In the following sentence, what does “impact” mean in this context?

 The sentence "While some results of stakeholder and citizen engagement activities were adopted, recommendations were not always translated into policies.” It can be shortened, as the first subsentence says the same as the second subsentence.

**Conclusion**

 This is too brief. Can you add the implications of this finding? Is this good, bad, or relevant for what?

 Please add a conclusion on all three research questions that you have posed in the article.

**Introduction**

 When you start to introduce Climate Assemblies, you use the term “deliberative mini publics”. This is confusing. I would rather start with calling them by the term that you use throughout the paper (Climate Assemblies) and later mention that they are also called “deliberative mini publics”.

 Separate the information that relates to deliberate democracy in general and to Climate Assemblies in particular. I.e., put all the info to Climate Assemblies into a separate paragraph.

 Separate the research questions into a separate paragraph.

 A full stop is missing in the paragraph before the RQs.

 Policy-making and policymaking: please harmonize and use one writing in the article.

 Last paragraph before “Methods” in one sentence you say “the next section”, and in the subsequent sentence you say "the following section”. One could misread this as both pointing to the next section, i.e. the Methods. Please revise.

 Overall, this last paragraph before the Methods needs to be reworked. It mixes information on methods, data, and results, as well as how it is presented.

**Methods**

 Put “first research question” in brackets, as you have done for RQ2 and RQ3.

 “In most cases”, or “in a few instances”, or “in a few cases”: it would read better in a research paper if you were more specific and less descriptive. Please add specifics.

**Stakeholder workshops**

 Drivers and barriers (D&B): Do you mean D&B to the formation of CAs? Or to the participation in CAs? Or to what?

**Survey**

 I believe the abbreviation CLIMAS has not been explained before.

 Could you add the survey as Supplementary information, please?

**Results**

 The sentence “CAs included were more prevalent in some countries than in others.” Can be excluded. This is almost always the case. It does not add substantial information.

 “This paper focuses on…” should be at the beginning of a paragraph; otherwise, it appears subordinate. You would bury the most important somewhere within a paragraph.

 Please put the results regarding % of those who initiated a CA first. Then add what the different forms of initiation imply.

 Regarding “non-existent”, “weak”, “close” or “fixed” connections, please explain what each of those means, in particular “close” and “fixed”… and how you distinguish between these different forms.

 “Do they consider them part of their self-perception?”: Who is “they”?

 There are differences between CAs with an interest in politics and those without. You say that the latter case was observed for CAs on transport and air pollution. What were the characteristics of those in which politicians showed interest?

**Stakeholder workshops on Drivers**

 The methodology section mentions that both the stakeholder workshops and a survey were used to elicit drivers and barriers. But here, you only report on the Stakeholder workshops? Then, that part in the methodology can be deleted/ modified. Or do you combine data from both the workshops and the survey in the results section on drivers? And, if not, why do you only report those from the workshop?

 I don’t understand why there are two lists of important drivers; 1) as mentioned in this sentence, “Important drivers identified in the workshops included measures to safeguard equality and inclusiveness in access, the relevance of the CA to citizens, and its impact.” And 2) as highlighted in these paragraphs of drivers, i.e. “Importance of simple language”, “Offering financial incentives”… please explain in the paper. The most important ones should be explained in detail, the somewhat less important one could be mentioned in a list. …..

 The same applies to the barriers section (there is a sentence with a list, and the paragraphs on barriers, which do not align).

 From what I read in the text, it seems that “Offering financial incentives” is not an important driver, but it received mixed outcomes. Why is it highlighted then?

 Similarly, the “involvement” of journalists was not uniformly perceived as a driver, or?

 Overall, in the Results section or the Methodology, I am missing a map of the spatial distribution of the data and results (for the various methods reported).

**Discussion**

 There are various locations/sections in the “Results” section that actually belong in the discussion. Please review the Results and Discussion section carefully and move the relevant parts to the proper sections.

 Somewhere in the discussion, the potential limitations of the workshop and survey, e.g., the survey's low sample size, and other potential limitations should be mentioned.

If applicable, is the statistical analysis and its interpretation appropriate?

Yes

Is the study design appropriate and is the work technically sound?

Yes

Is the work clearly and accurately presented and does it engage with the current literature?

Yes

Are the conclusions drawn adequately supported by the results?

Yes

Are sufficient details of methods and analysis provided to allow replication by others?

No

Are all the source data and materials underlying the results available?

No

Reviewer Expertise:

Climate Change Planning and Governance

I confirm that I have read this submission and believe that I have an appropriate level of expertise to confirm that it is of an acceptable scientific standard, however I have significant reservations, as outlined above.

# Reviewer report: Reviewer response for version 1

**DOI:** 10.21956/openreseurope.23203.r67566

**Published:** 2026-01-20

**Authors:** Anna Boqué-Ciurana

**Affiliations:** 1 Rovira i Virgili University, Tarragona, Catalonia, Spain

Citizen participation in climate politics: Drivers and barriers of climate assemblies in Europe

**Background and overall structure**

 The background is clearly presented and situates the study well within current debates on climate assemblies in Europe. The research questions are appropriate and clearly formulated, and the plain language summary is clear and accessible. The methodology is robust and well explained, and the results, discussion and conclusion are generally well structured and presented.

**Introduction**

- The introduction is well written, but it would be valuable to include more reflection on the **profile** of participants in climate assemblies, especially in relation to age.
- You could explicitly address whether young people and children are included in these assemblies and, if not, discuss the implications of their absence.
- It would strengthen the introduction to briefly mention approaches that have specifically involved young people in citizen assemblies, for example:
  - **Ref 1 : (Harris, 2021)**
  - **Ref 2: (Cebrián et al., 2025)**
- A short paragraph connecting these experiences with your focus on European climate assemblies would underline the importance of intergenerational justice and the inclusion of younger voices.

**Methodology**

- The methodology section is solid and clearly explained. To further enhance clarity and transparency, it would be very helpful to add a **flow diagram** summarising the methodological design and the sequence of the three methods used (e.g. document analysis, survey, workshops).
- This diagram could show, in a single visual, the different steps, the number and type of cases/participants at each step, and how insights from each method inform the next.

**Survey section**

- In the description of the survey, you should include more precise information on the **statistical characteristics** of the data.
- Consider adding:
  - Information on statistical significance for key comparisons or associations you report (e.g. p-values, confidence intervals, or effect sizes when relevant).
- This will increase the robustness and credibility of the quantitative part of your analysis.

**Results**

- The first part of the results is clearly presented and logically structured.
- The subsection on the stakeholder workshop on drivers and barriers of climate assemblies is also well explained and informative.
- For Figures 6 and 7, it would be very useful to add a **colour legend** explicitly indicating what green, yellow, orange and grey represent. This will make the figures self-explanatory and easier to interpret for readers who may skim the text.

**Discussion and conclusion**

- The discussion appropriately connects the findings to the broader literature and policy implications, and the conclusions are clear and consistent with the results.
- Once you include the elements suggested above (youth participation, methodological diagram, survey statistics, figure legends), you might add one or two sentences in the discussion acknowledging these aspects as strengths of your approach and/or pointing to them as recommendations for future practice and research on climate assemblies.

If applicable, is the statistical analysis and its interpretation appropriate?

Partly

Is the study design appropriate and is the work technically sound?

Partly

Is the work clearly and accurately presented and does it engage with the current literature?

Partly

Are the conclusions drawn adequately supported by the results?

Partly

Are sufficient details of methods and analysis provided to allow replication by others?

Partly

Are all the source data and materials underlying the results available?

Yes

Reviewer Expertise:

Climate Services, Climatology, Climate School Assemblies

I confirm that I have read this submission and believe that I have an appropriate level of expertise to confirm that it is of an acceptable scientific standard.

## References

[1] : Looking to the future? Including children, young people and future generations in deliberations on climate action: Ireland's Citizens’Assembly 2016–2018. *Innovation: The European Journal of Social Science Research* .2021;34(5) : 10.1080/13511610.2021.1968356 677-693 10.1080/13511610.2021.1968356

[2] : School climate assemblies: an educational tool for empowering pupils and youth to take climate and sustainability action. *Sustainability Science* .2025;20(1) : 10.1007/s11625-024-01583-6 135-153 10.1007/s11625-024-01583-6
